# m^6^A eraser ALKBH5 mitigates the apoptosis of cardiomyocytes in ischemia reperfusion injury through m^6^A/SIRT1 axis

**DOI:** 10.7717/peerj.15269

**Published:** 2023-05-11

**Authors:** Liangliang Liu, Zhen Liu

**Affiliations:** Department of Cardiology, Lianyungang Hospital of Traditional Chinese Medicine, Lianyungang, China

**Keywords:** ALKBH5, Cardiomyocytes

## Abstract

Recent studies have shown that the potential regulatory role of N^6^-methyladenine (m^6^A) modification may affect the occurrence and development of various cardiovascular diseases. However, the regulatory mechanism of m^6^A modification on myocardial ischemia reperfusion injury (MIRI) is rarely reported. A mouse model of myocardial ischemia reperfusion (I/R) was established by ligation and perfusion of the left anterior descending coronary artery, and a cellular model of hypoxia/reperfusion (H/R) was conducted in cardiomyocytes (CMs). We found that the protein expression of ALKBH5 in myocardial tissues and cells were decreased, accompanied by increased m^6^A modification level. Overexpression of ALKBH5 significantly inhibited H/R-induced oxidative stress and apoptosis in CMs. Mechanistically, there was an enriched m^6^A motif in the 3′-UTR of SIRT1 genome, and ALKBH5 overexpression promoted the stability of SIRT1 mRNA. Furthermore, results using overexpression or knockdown of SIRT1 confirmed the protective effect of SIRT1 on H/R induced CMs apoptosis. Together, our study reveals a critical role of ALKBH5-medicated m^6^A on CM apoptosis, supplying an important regulating effect of m^6^A methylation in ischemic heart disease.

## Introduction

Myocardial ischemia-reperfusion injury (MIRI) is considered to be one of the main causes of death in patients with acute myocardial infarction and coronary heart disease (CHD). How to restore the blood flow of ischemic tissue as early as possible and relieve the damage caused by reperfusion at the same time has become the key issue to the treatment of ischemic heart disease (Fröhlich et al., 2013).

Abundant evidence shows that MIRI is closely related to cardiomyocyte apoptosis. The main manifestation of cell death in the acute phase of myocardial infarction is apoptosis, so early inhibition of myocardial cell apoptosis can significantly improve MIRI. As a member of NAD+ dependent sirtuin deacetylases, Sirtuin-1 (SIRT1) catalyzes the deacetylation of histone proteins as well as other substrates, and participates in pathological changes such as oxidative stress, inflammation, apoptosis, mitochondrial energy metabolism, DNA damage, and autophagy. On the contrary, SIRT1 deficiency leads to increased oxidative stress, inflammation, apoptosis and autophagy, promoting blood vessels aging and atherosclerosis (Sosnowska et al., 2017). During myocardial I/R, SIRT1 attenuates inflammation and pyroptosis *via* Akt-dependent metabolic regulation (Han et al., 2020).

In recent years, more and more studies have confirmed that RNA methylation modification is involved in the occurrence and development of cardiovascular diseases (CVD) (Qin et al., 2020). N^6^-methyladenine (m^6^A) methylation, as the main form of RNA methylation modification, are dynamically regulated with specified writers (methyltransferases) that catalyze addition of m^6^A (METTL3, METTL4, METTL14, and WTAP), specified erasers (demethylases) that catalyze removal of m^6^A (FTO, ALKBH5) from mRNA, and variable reader (YTHDFs and IGF2BPs) to develop different downstream effects (Jiang et al., 2021). M^6^A might act as a molecular switch to regulate the splicing, stability, and positioning of mRNA (Fu et al., 2014). The whole genome m^6^A sequencing analysis proved the occurrence of m^6^A in cardiomyocytes, and it will dynamically change under hypertrophic conditions (Xu et al., 2022). Further research showed that METTL3 affects cardiomyocyte hypertrophy and induces spontaneous myocardial remodeling by controlling gene expression programs, suggesting that METTL3 may be a potential therapeutic target for pathological cardiac remodeling (Dorn et al., 2019). Besides, m^6^A modification was found to be significantly enhanced in failed mammalian hearts, accompanied by decreased alpha-ketoglutarate dependent dioxygenase (FTO) myocardial contractile dysfunction, while overexpression of FTO reduced myocardial fibrosis and increased angiogenesis (Mathiyalagan et al., 2019). Another demethylase α-ketoglutarate dependent dioxygenase alkB homolog 5 (ALKBH5), after being induced to overexpression, significantly promotes mitosis and regeneration of primary cardiomyocytes, and improves cardiac function after ischemic injury (Han et al., 2021).

This study constructed the mice myocardial I/R model and H/R cardiomyocytes, and found the anomalously expressed m^6^A modification and ALKBH5, which involved in cardiomyocytes apoptosis. Furthermore, we explored the role of ALKBH5 in the apoptosis of cardiomyocytes treated with ischemia reperfusion (I/R) and addressed the mechanism by which m^6^A participates in the MIRI.

## Materials and Methods

### Establishment of mice I/R model

Male C57BL/6 mice (25–30 g) were obtained from Vital River Laboratory Animal Technology (Beijing, China) and were provided adaptive feeding for a week at the suitable temperature and humidity. All animals were housed in micro-isolator cages with free access to food and water according to the Guide for the Care and Use of Laboratory Animals. The myocardial I/R operation were followed by previous research (Song et al., 2015). The mice were randomly divided into myocardial I/R group (*n* = 10) and sham group (*n* = 10). Mice were anesthetized (50 mg/kg pentobarbital sodium, intraperitoneal injection) before assays. The supine of mice were fixed on the operating table connected with the standard lead II electrocardiogram. The left thorax was cut to expose the heart and the left anterior descending (LAD) coronary artery was ligated by 7/0 sterile suture. Myocardial ischemia was induced by LAD ligation for 30 min followed by 120 min of reperfusion. Sham group mice underwent the same surgical procedures without LAD coronary artery ligation. After assay, the surviving animals were transferred to institution’s animal department for euthanizing mice. The project was approved by the Ethics Committee of Lianyungang Hospital of Traditional Chinese Medicine (No. LYGH2018026).

### Primary cardiomyocytes (CMs) culture and hypoxia/reperfusion (H/R) injury administration

Primary neonatal ventricular myocytes were acquired by enzymatic digestion of 1–4 days old neonatal mice hearts as described (Han et al., 2021). The supernatant of each round of cell digestion and cultured with DMEM medium (Hyclone, Logan, UT, USA) supplemented with 10% fetal bovine serum (FBS) and 1% penicillin-streptomycin liquid in 37 °C incubator containing 5% CO_2_. After centrifugation to remove the supernatant, the cells are resuspended and cultured in a culture flask. To mimic hypoxia/reperfusion (H/R), CMs were treated with 4 h of hypoxia (1% O_2_, 5% CO_2_, 94% nitrogen) and then followed by 3 h reoxygenation (5% CO_2_, 21% O_2_, 74% nitrogen) as described previously (Song et al., 2015).

### Plasmids and cell transfection

The ALKBH5-overexpression plasmids (pcDNA3.1-ALKBH5), vector controls (pcDNA.3.1-NC) and small interfering RNAs (siRNAs) (si-ALKBH5-1, si-ALKBH5-2 and si-NC) were designed and synthesized from GemmaPharma (Shanghai, China). Transfection of siRNAs and plasmids was performed using Lipofectamine 2000 (Thermo Fisher Scientific, Waltham, MA, USA) according to the manufacturer’s instructions. Cells were harvested at 48 h for future analysis.

### Western blotting

Cells total protein was extracted by RIPA lysis buffer after different treatments with protease inhibitor (Solarbio, Beijing, China), and protein concentration were quantified by BCA protein detection kit (Solarbio, Beijing, China). The proteins were separated using SDS-PAGE (Solarbio, Beijing, China) and transferred to the PVDF membranes (Millipore, Burlington, MA, USA) by an electroblot apparatus. Membranes were incubated at 4 °C overnight with primary antibodies of ALKBH5 (1:1,000, ab195377; Abcam, Cambridge, UK), Sirt1 (1:1,000, ab189494; Abcam, Cambridge, UK) and GAPDH (1:1,000; Cell Signaling Technology, Danvers, MA, USA). After being washed three times with 0.1% TBST, membranes were incubated with secondary antibody (1:1,000; Cell Signaling Technology, Danvers, MA, USA) for 2 h at room temperature. Bands were visualized with an enhanced chemiluminescence (ECL) detection reagent (Millipore, Burlington, MA, USA). The intensity of the bands was quantified using Image J software.

### Quantitative real-time PCR

Total RNA was separated from myocardial tissues or CMs using Trizol reagent (Invitrogen, Carlsbad, CA, USA), and then cDNA was synthesized using Transcriptor First Strand cDNA Synthesis Kit (Roche, Indianapolis, IN, USA). Real-time PCR was performed using SYBR Green PCR Master Mix (Takara, Dalian, China) on Applied Biosystems 7500. Amplification was performed as follows: a denaturation step at 94 °C for 5 min, followed by 40 cycles of amplification at 95 °C for 30 s, 60 °C for 32 s and 72 °C for 30 s. The relative expression levels were calculated by 2^–ΔΔCt^ method, and normalized to GAPDH mRNA. The primers were listed in Table S1.

### Flow cytometric analysis

Cell apoptosis were analyzed by flow cytometric analysis. Briefly, CMs were collected after transfection 48 h, and incubated with Annexin V (BD Biosciences, San Jose, CA, USA) for 15 min. Then, 5 μl Annexin-FITC were added and incubated for 15 min at dark room temperature. Apoptosis rate were generated using flow cytometry with Modifit software (Verity Software House, Topsham, ME, USA).

### Enzyme-linked immunosorbent assay (ELISA)

The markers of myocardial injury creatine kinase (CK) and lactic dehydrogenase (LDH), and oxidative stress related indicators cytoplasmic form superoxide dismutase (SOD), malonaldehyde (MDA) and glutathione peroxidase (GSH-px) were detected by ELISA. After treated with indicated processing, cells supernatants were collected to detect concentration of cytoplasmic form SOD, MDA and GSH-px on the basis of the manufacturer’s instructions.

### RNA stability assay

CMs were seeded in six-well plates (1 × 10^5^ cells per well) for 24 h. Then actinomycin D (Act D; Sigma, St. Louis, MI, USA) was added to 2 μg/ml at indicated time (0, 3 and 6 h) before cell collection. RNA was extracted and real-time PCR were performed as described earlier.

### Total m^6^A quantification

The total m^6^A mRNA levels were determined using an m^6^A methylation quantification kit (EpiGentek, Farmingdale, NY, USA) according to the manufacturer’s protocol. Briefly, total RNA was extracted and coated on assay wells (200 ng per well). After that, m^6^A antibody in solution was added into each well. The m^6^A levels were detected at wavelength of 450 nm absorbance.

### MeRIP-qPCR

Total RNA were isolated from CMs after ALKBH5 overexpression or knockdown, and treated with genomic DNA purification reagents (Sigma, St. Louis, MI, USA). After fragmentation, the mRNA was incubated with primary antibody of m^6^A with a Magna MeRIP™ m^6^A kit (#17-10499; Millipore, MA, USA). The m^6^A bound RNA was then detected through qRT-PCR.

### RNA immunoprecipitation assay

RNA immunoprecipitation (RIP) was performed using Magna RIP™ RNA-Binding Protein Immunoprecipitation Kit (Millipore, Burlington, MA, USA) according to the manufacturer’s protocol. CMs were collected and lysed in complete RIP buffer comprising protease inhibitor cocktail and RNase inhibitor. Anti-m^6^A antibody or anti-ALLKBH5 antibody was incubated with RIP buffer containing magnetic bead conjugated with indicated antibody or control IgG for 1.5 h at 4 °C. The purified RNA was subjected to qRT-PCR to determine the binding target.

### Analysis of publicly available datasets

SRAMP (http://www.cuilab.cn/sramp) tools was utilized to predicting potential m^6^A modification sites of SIRT1 mRNA.

### Statistical analysis

All the experiments were performed three times at least. The data were expressed as the mean ± SD SPSS software (version 18.0; IBM Corp., Armonk, NY, USA) and GraphPad Prism (version 8.0; GraphPad Software, La Jolla, CA, USA) were used to analysis the statistical results. Differences between groups were estimated using a *t*-test. The comparisons of multiple groups were performed by one-way ANOVA and then an LSD-t test. *P* < 0.05 were considered statistically significant.

## Results

### I/R induces m^6^A demethylase ALKBH5 downregulation in CMs

**Figure 1:**
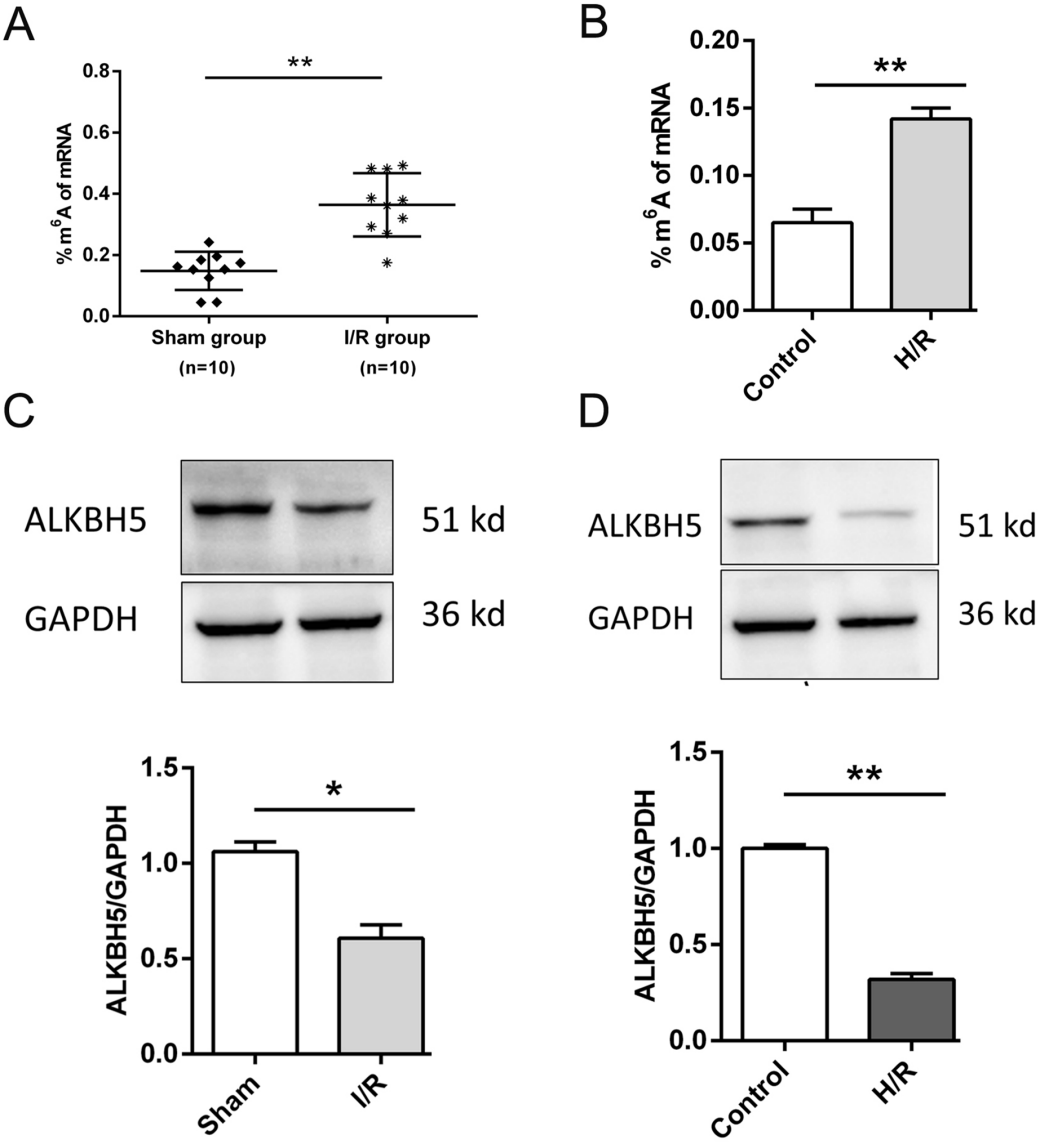
M^6^A demethylase ALKBH5 is down-regulated in myocardial I/R vivo and vitro. (A) M^6^A modification level was detected in I/R myocardial tissues by colorimetric method, *n* = 10 and *n* = 10. (B) CMs were treated with hypoxia reperfusion and m^6^A level was detected using colorimetric method, *n* = 3. (C and D) The protein expression of ALKBH5 in myocardial (C) tissues and (D) cells were detected by western blot assay, *n* = 3. Error bars represent the SD obtained from at least three biological replicates. *P < 0.05, **P < 0.01.

As shown in Fig. 1, we established the mice myocardial I/R injury and hypoxia/reperfusion (H/R) models *in vivo* and *vitro*. M^6^A quantification analysis found that m^6^A methylation was significantly increased in I/R group and H/R group (Figs. 1A and 1B). Then, total protein was extracted to detect the expression of ALKBH5 by western blotting. Results indicated that ALKBH5 was significantly down-regulated in I/R myocardium and H/R induced CMs (Figs. 1C and 1D). These findings suggested that ALKBH5 mediated m^6^A modification changes may play a vital role in myocardial I/R injury.

### ALKBH5 overexpression attenuates H/R induced CM apoptosis

**Figure 2:**
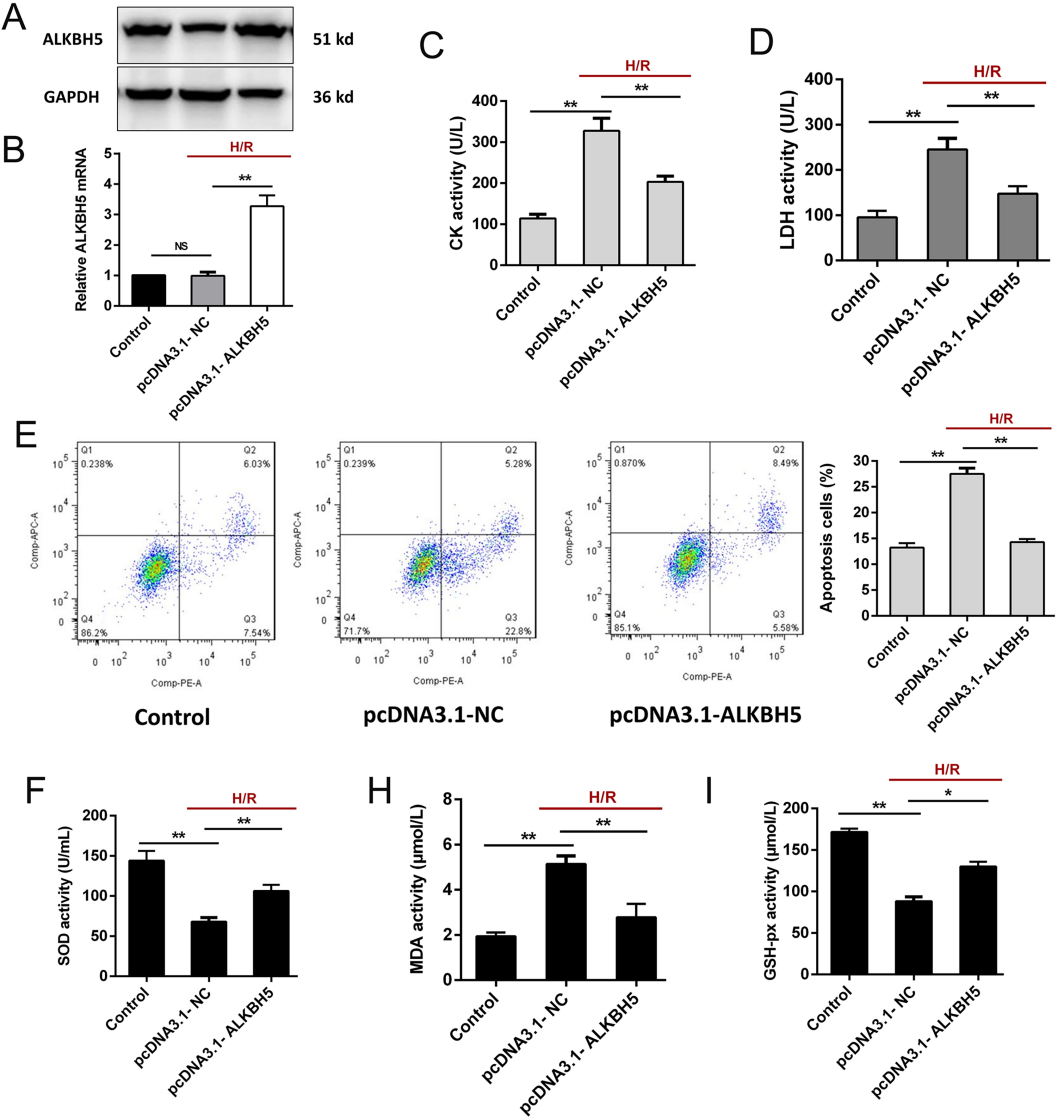
Overexpression of ALKBH5 inhibits H/R induced CMs apoptosis. (A) Transfection efficiency of ALKBH5 was detected using western blotting and (B) qRT-PCR. GAPDH was the internal reference. (C) CK activity and LDH activity (D) were assessed for the cell injury degree in ALKBH5 overexpression cell lines. (E) Flow cytometry assay was used to confirm the apoptosis analysis induced by overexpression of ALKBH5, *n* = 3. (F) The alterations of oxidative stress-associated enzymes SOD (cytoplasmic form), MDA and GSH-px activity were all assessed by ELISA assay, *n* = 3. Error bars represent the SD obtained from at least three biological replicates. *P < 0.05, **P < 0.01.

To investigate the function of ALKBH5 in regulating CMs, we transfected the plasmid encoding ALKBH5 (pcDNA3.1-NC and pcDNA3.1-ALKBH5) into CMs and detecting the transfection efficiency using qRT-PCR and western blotting. As shown (Figs. 2A and 2B), the expression of ALKBH5 mRNA and protein were significantly up-regulated in upon overexpression. Besides, H/R stimulation increased the release of CK and LDH, and overexpression of ALKBH5 obviously reduced CMs injury (Figs. 2C and 2D). Apoptosis analysis using flow cytometry showed that ALKBH5 overexpression decreased apoptosis cells (Fig. 2E). An ELISA assay further demonstrated that ALKBH5 overexpression significantly increased antioxidases cytoplasmic form SOD and GSH-px concentration, and decreased the concentration of membrane lipid peroxidation product MDA (Figs. 2F–2H). These results indicated that ALKBH5 overexpression attenuates H/R induced CMs apoptosis.

### ALKBH5 increased SIRT1 mRNA stability *via* inhibiting its m^6^A level

**Figure 3:**
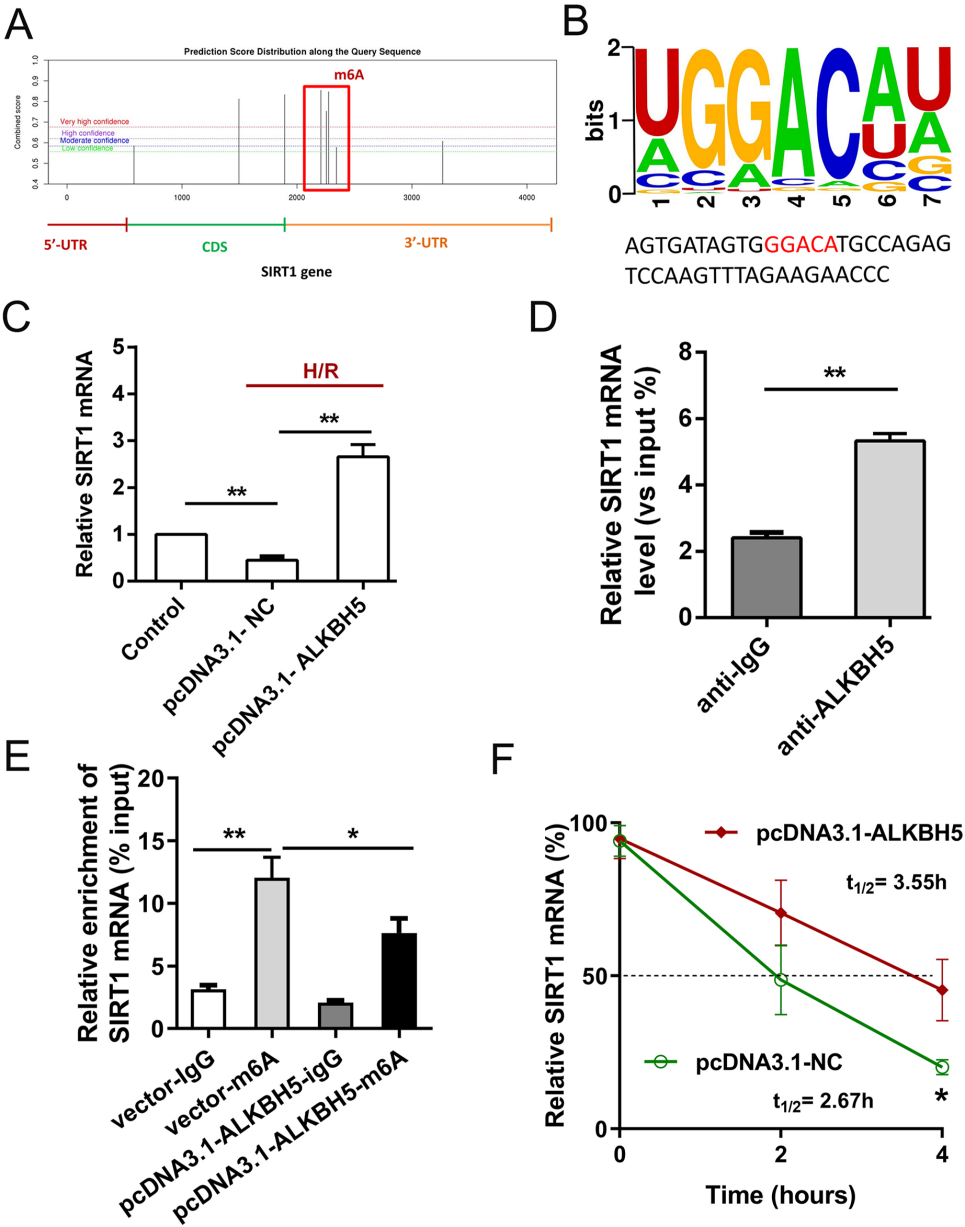
ALKBH5 increase SIRT1 mRNA stability *via* m^6^A dependent manner. (A) The potential m^6^A sites in the 3′-UTR with vary high possibility predicted by SRAMP online tool. (B) The m^6^A binding sites or modify of ALKBH5 were GGACA. (C) The mRNA level of SIRT1 was analyzed by qRT-PCR after CMs transfected with plasmids of ALKBH5, *n* = 3. (D) RIP-PCR was performed to confirm the SIRT1 mRNA enrichment by anti-ALKBH5 antibody in CMs, *n* = 3. (E) MeRIP-qPCR was used to assess the m^6^A level of SIRT1 mRNA after overexpressing ALKBH5 in CMs, *n* = 3. (F) The mRNA stability and degradation halftime of SIRT1 in CMs treated with Actinomycin D, *n* = 3. Error bars represent the SD obtained from at least three biological replicates. *P < 0.05, **P < 0.01.

Previous study showed that SIRT1 deficiency participated in inflammation, oxidative stress, autophagy and impaired nitric oxide production, thereby promoting atherosclerosis and I/R (Sosnowska et al., 2017). In metabolic differences between the neonatal heart and adult heart, SIRT1 can vary from stress and age. In this study, we tried to explore whether there is an molecular interaction within ALKBH5 and SIRT1 in MIRI processes using SRAMP (http://www.cuilab.cn/sramp). SRAMP combines three random forest classifiers that exploit the positional nucleotide sequence pattern, the K-nearest neighbor information and the position-independent nucleotide pair spectrum features. SRAMP uses either genomic sequences or cDNA sequences as its input. Thus, SRAMP acts as a powerful tool to predict the m^6^A site on genes. We used the SRAMP online prediction tool to find that there were several potential and very high possibility m^6^A modification sites in the SIRT1 3′-UTR (Fig. 3A). Interestingly, we identified that the m^6^A modification sequence ‘GGACA’ on SIRT1 mRNA is basically the same as the modification sites of ALKBH5 (Fig. 3B). To verify our predictions, RT-qPCR assay and RIP assay were performed, and the results showed that ALKBH5 overexpression significantly up-regulated SIRT1 mRNA expression (Fig. 3C). Meanwhile, SIRT1 mRNA level was significantly enriched by anti-ALKBH5 antibody in immunoprecipitation (Fig. 3D), and ALKBH5 overexpression repressed the m^6^A modification level of SIRT1 mRNA in anti-ALKBH5 immunoprecipitation (Fig. 3E). Moreover, we analyzed SIRT1 mRNA degradation by qRT-PCR in actinomycin D treated CMs. The result showed that ALKBH5 overexpression significantly decreased the degradation of SIRT1 mRNA (Fig. 3F), which means ALKBH5 could increase the stability of SIRT1 mRNA *via* inhibiting its m^6^A level.

### SIRT1 regulated H/R induced oxidative stress and apoptosis

**Figure 4:**
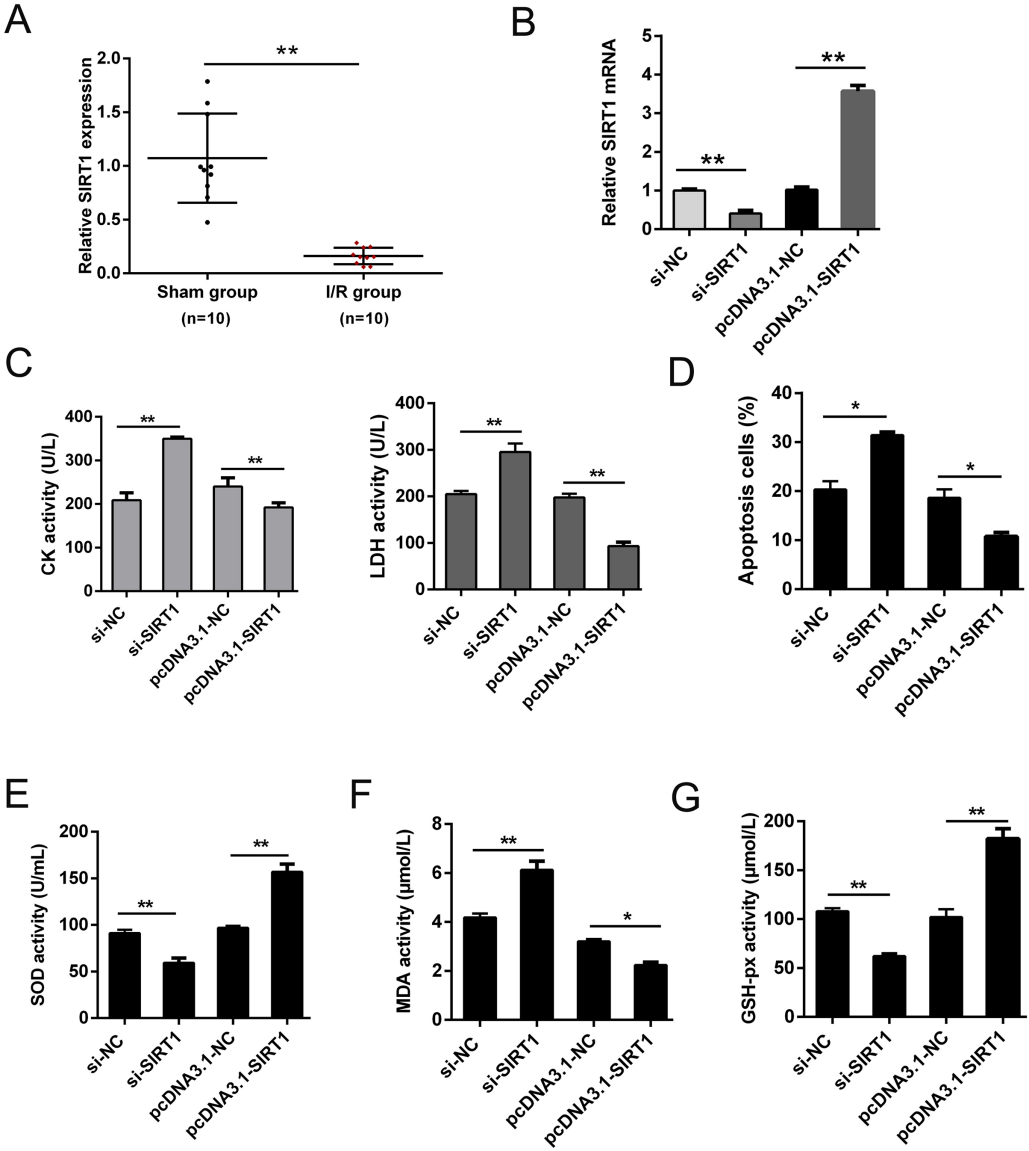
SIRT1 positively improves I/R-induced CMs apoptosis. (A) The mRNA expression of SIRT1 in myocardial I/R tissues, *n* = 10. (B) Knockdown and overexpression efficiency of SIRT1 in H/R induced CMs were detected by qRT-PCR, *n* = 3. (C) CK and LDH activity were measured after CMs transfected with siRNAs or plasmids, *n* = 3. (D) Flow cytometry assay was performed to assess the apoptosis rate after knockdown or overexpress SIRT1. Apoptotic cells were calculated and presented by histogram, *n* = 3. (E) Cytoplasmic form SOD activity. (F) MDA activity, *n* = 3. (G) GSH-px activity, *n* = 3. Error bars represent the SD obtained from at least three biological replicates. *P < 0.05, **P < 0.01.

SIRT1 has been reported modulates cardiac metabolism that medicated inflammatory response during I/R stress (Han et al., 2020; Wang et al., 2018). In this study, we detected the expression of SIRT1 mRNA *in vivo* I/R models. As shown in Fig. 4A, SIRT1 mRNA was obviously down-regulated in I/R group. For further clarify the effect of SIRT1 on H/R CMs, siRNAs or plasmids were transfected into CMs. The transfection efficiency was detected using qRT-PCR (Fig. 4B). Knockdown of SIRT1 showed significantly effect of promoting CK and LDH release, and overexpression of SIRT1 indicated opposite effect (Fig. 4C). Moreover, flow cytometry suggested that apoptosis could be aggravated by knocking down SIRT1 (Fig. 4D). Oxidative stress-associated enzymes were measured by ELISA assay, and the results showed that cytoplasmic form SOD and GSH-px activity were suppressed, and MDA activity was growing by silencing SIRT1, while the opposite results were shown by overexpress SIRT1 (Fig. 4E). Above results indicated that SIRT1 can regulate H/R induced cardiomyocyte oxidative stress and apoptosis.

## Discussion

MIRI is the major cause of ischemic heart disease and post-ischemic key pathological processes in cardiac remodeling (Liu et al., 2022). Therefore, strategies to clarify the mechanisms of myocardial injury after reperfusion are particularly important (Wu et al., 2021). In this study, we found that the m^6^A eraser ALKBH5 was significantly down-regulated in I/R model of *in vivo* and *vitro*. Overexpression of ALKBH5 remarkably inhibited CM apoptosis, oxidative stress and release of myocardial enzymes. Mechanistically, ALKBH5-mediated m^6^A demethylation increased stability of SIRT1 mRNA and promoted the expression of SIRT1, thus motivating the translation of SIRT. Moreover, we proved that forced SIRT1 overexpression led to a significant decrease of myocardial enzymes and apoptosis. These findings expand our view that ALKBH5-dependent m^6^A modification may be a potential target for the treatment of I/R-related ischemic heart disease.

Recent studies have shown that m^6^A RNA methylation is closely related to pathological stimuli in MIRI, such as oxidative stress, inflammation, autophagy and apoptosis (Qin et al., 2020; Zhao et al., 2020). Compared with normal myocardial tissues, methyltransferase METTL3 is significantly up-regulated in myocardial tissues of patients with myocardial infarction, and promotes cardiomyocyte apoptosis under H/R by impairing autophagic flux, while demethylase ALKBH5 plays a opposite role in H/R mediated m^6^A modification of TFEB mRNA in cardiomyocytes (Song et al., 2019). Not only that, m^6^A modification played a role in anesthetic post-conditioning cardioprotection. Dexmedetomidine, a widely used anesthetic, was found reduces m^6^A methylation and attenuate cell death in H/R induced CM model (Peng et al., 2020). In addition, some researches uncovered a regulatory role of m^6^A modification in heart failure and myocardial hypertrophy. Han et al. (2021) reported that ALKBH5-mediated m^6^A regulates cardiomyocyte proliferation and regeneration by improving the stability of YTHDF1 and promoting the translation of YAP. Next-generation sequencing results uncovered that m^6^A landscape is altered in heart failure and heart hypertrophy, and leads to protein abundance changes independent of mRNA levels (Berulava et al., 2020). These findings enhance our understanding that the relationship between m^6^A modification and origin of cardiovascular disease.

The important roles of SIRTs in many physiological processes and disease pathologies are being investigated, including cancer, diabetes, and cardiovascular disease. SIRT1 has been involved in protection against myocardial infarction and reperfusion injury, endothelial dysfunction and thrombosis (Meng et al., 2017). Moreover, increased expression of SIRT1 can reduce the infarct size, implying a protective role of SIRT1 on the myocardium (Shalwala et al., 2014). The results of this study are in accordance with reported studies, that is, the expression of SIRT1 is reduced in H/R-induced CMs, and overexpression of SIRT1 significantly inhibits oxidative stress and apoptosis of CMs under the H/R condition. More than that, downregulation of SIRT1 expression was observed in hippocampus and liver I/R injury, whereas administration of resveratrol or ischemic preconditioning significantly inhibited oxidative stress and activating SIRT1 (Singh & Ubaid, 2020). Therefore, SIRT1 may serve as a protective target against multiply organ I/R injuries.

However, there are some limitations in this study. The dynamic evolution of m^6^A modifications is regulated by writers, erasers and readers. Our study focuses on the regulation of SIRT1 mRNA stability by ALKBH5-mediated m^6^A modification, and it is unclear whether there are other writers or erasers co-regulating roles. In addition, the reader is the protein that actually exerts the m^6^A modification instruction, and its action in this study remains to be further clarified. Finally, the interplay among SIRT1 and its downstream targets including NF-κB, PGC-1α, FOXO *etc*. depict complex molecular networks that protect the tissues from I/R induced injury. Future studies should verify the downstream targets of SIRT1 under I/R conditions.

**Figure 5:**
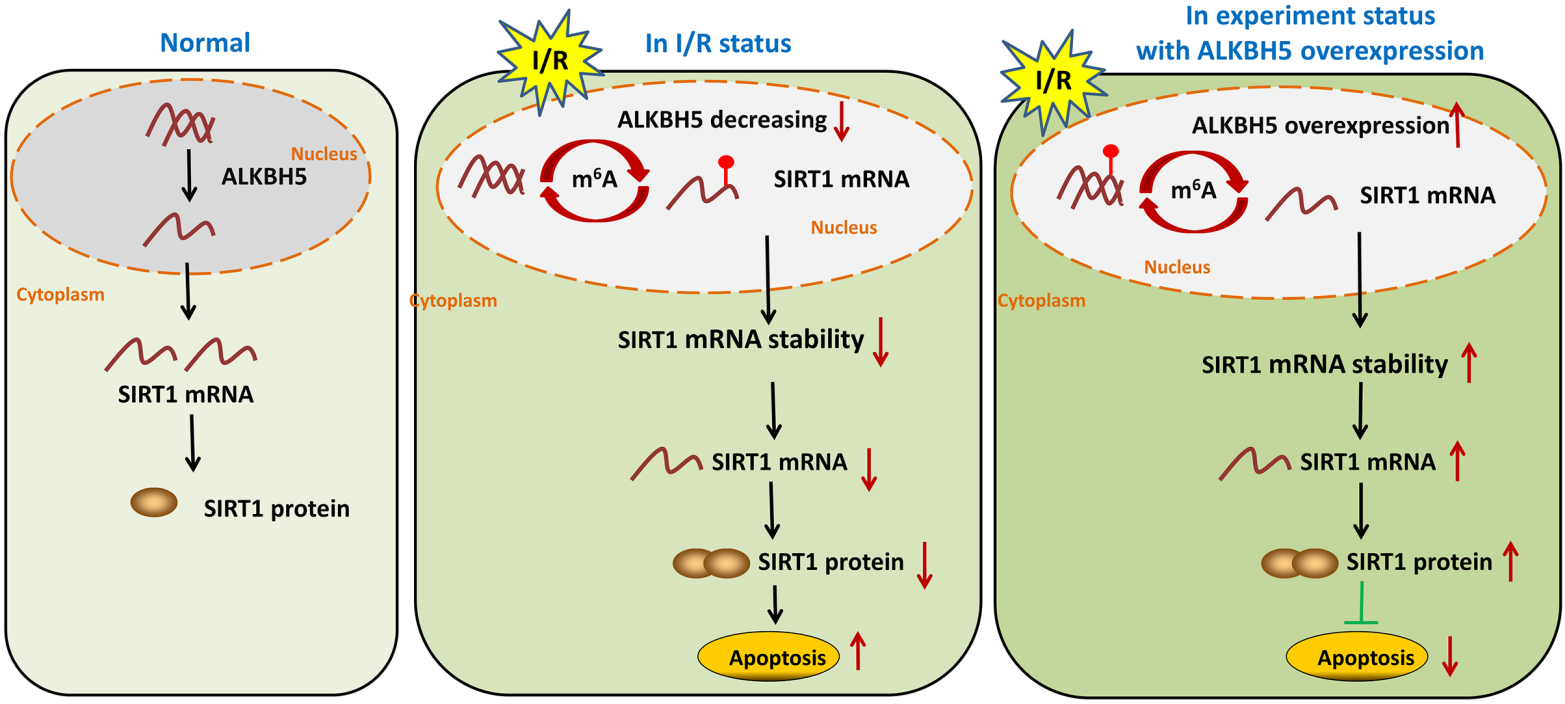
m^6^A demethylase ALKBH5 regulated myocardial I/R-induced CMs apoptosis *via* increasing stability of SIRT1 mRNA. This study demonstrated m^6^A demethylase ALKBH5 regulated myocardial I/R induced CMs oxidative stress and apoptosis *via* increasing stability of SIRT1 mRNA. The results may enrich the pathogenesis of MIRI, and provide a potential therapeutic target for myocardial I/R injury.

In summary, our study demonstrated demethylase ALKBH5 regulated myocardial I/R induced CMs oxidative stress and apoptosis *via* increasing stability of SIRT1 mRNA (Fig. 5). The results may enrich the pathogenesis of MIRI, and provide a potential therapeutic target for myocardial I/R injury.

## Supplemental Information

10.7717/peerj.15269/supp-1 Supplemental Information 1 Author Checklist.

10.7717/peerj.15269/supp-2 Supplemental Information 2 The primer sequences of the related genes.

10.7717/peerj.15269/supp-3 Supplemental Information 3 Blot images.

10.7717/peerj.15269/supp-4 Supplemental Information 4 Raw data including WB, PCR, ROS, LDH *etc*.

10.7717/peerj.15269/supp-5 Supplemental Information 5 Figure 3 PCR.

10.7717/peerj.15269/supp-6 Supplemental Information 6 Figure 4 PCR.

## Additional Information and Declarations

### Data Availability

The following information was supplied regarding data availability: The raw data are available in the Supplemental File.
