# Supplementary material for: m6A eraser ALKBH5 mitigates the apoptosis of cardiomyocytes in ischemia reperfusion injury through m6A/SIRT1 axis
Source: PeerJ. 2023 May 11;11:e15269. doi: 10.7717/peerj.15269 (PMC10183170; doi:10.7717/peerj.15269)
Supplement: Supplemental Information 2 [file peerj-11-15269-s002.docx]

Table S1. The primer sequences of the related genes.

| Gene | Sequence of primers (5’-3’) |
| --- | --- |
| ALKBH5 | Forward: 5’-CCCGAGGGCTTCGTCAACA-3’  Reverse: 5’-CGACACCCGAATAGGCTTGA-3’ |
| SIRT1 | Forward: 5’-CCGTGGCAAACTGGTACTTT-3’  Reverse: 5’-GACGCCAACATAGACCACCT-3’ |
| GAPDH | Forward: 5’-AGCCACATCGCTCAGACAC-3’  Reverse: 5’-GCCCAATACGACCAAATCC-3’ |
