# Supplementary figures and images for: m6A eraser ALKBH5 mitigates the apoptosis of cardiomyocytes in ischemia reperfusion injury through m6A/SIRT1 axis
Source: PeerJ. 2023 May 11;11:e15269. doi: 10.7717/peerj.15269 (PMC10183170; doi:10.7717/peerj.15269)

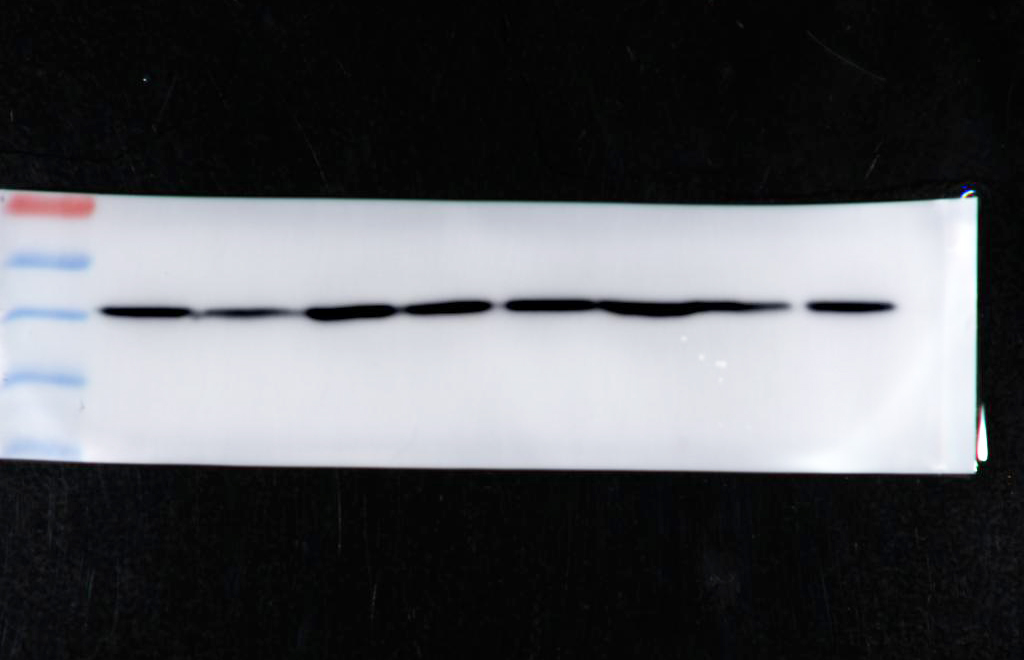

Supplement: Supplemental Information 3 [file peerj-11-15269-s003.zip › blots images/Fig 2-2 .jpg]

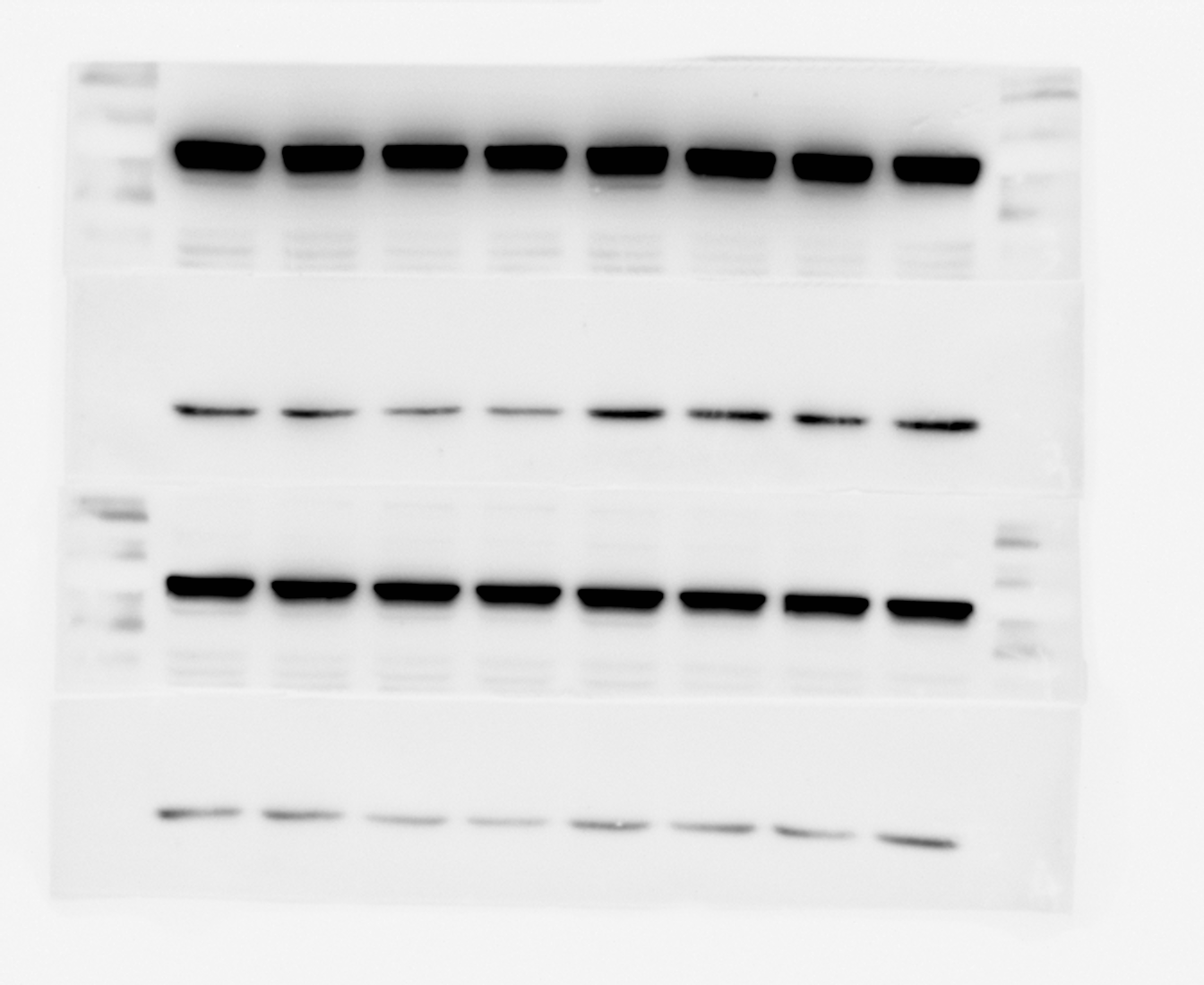

Supplement: Supplemental Information 3 [file peerj-11-15269-s003.zip › blots images/Fig 2.TIF]
